# Construction of a recombinant duck enteritis virus (DEV) expressing hemagglutinin of H5N1 avian influenza virus based on an infectious clone of DEV vaccine strain and evaluation of its efficacy in ducks and chickens

**DOI:** 10.1186/s12985-015-0354-9

**Published:** 2015-08-13

**Authors:** Jichun Wang, Aimin Ge, Mengwei Xu, Zhisheng Wang, Yongfeng Qiao, Yiqi Gu, Chang Liu, Yamei Liu, Jibo Hou

**Affiliations:** Jiangsu Academy of Agricultural Sciences/National Research Center of Veterinary Biologicals Engineering and Technology, Nanjing, 210014 China; Shandong Vocational Animal Science and Veterinary College, Weifang, 261061 China; College of Veterinary Medicine, Nanjing Agricultural University, Nanjing, 210095 China

## Abstract

**Background:**

Highly pathogenic avian influenza virus (AIV) subtype H5N1 remains a threat to poultry. Duck enteritis virus (DEV)-vectored vaccines expressing AIV H5N1 hemagglutinin (HA) may be viable AIV and DEV vaccine candidates.

**Methods:**

To facilitate the generation and further improvement of DEV-vectored HA(H5) vaccines, we first constructed an infectious clone of DEV Chinese vaccine strain C-KCE (DEV^C-KCE^). Then, we generated a DEV-vectored HA(H5) vaccine (DEV-H5(UL55)) based on the bacterial artificial chromosome (BAC) by inserting a synthesized HA(H5) expression cassette with a pMCMV IE promoter and a consensus HA sequence into the noncoding area between UL55 and LORF11. The immunogenicity and protective efficacy of the resulting recombinant vaccine against DEV and AIV H5N1 were evaluated in both ducks and chickens.

**Results:**

The successful construction of DEV BAC and DEV-H5(UL55) was verified by restriction fragment length polymorphism analysis. Recovered virus from the BAC or mutants showed similar growth kinetics to their parental viruses. The robust expression of HA in chicken embryo fibroblasts infected with the DEV-vectored vaccine was confirmed by indirect immunofluorescence and western blotting analyses. A single dose of 10^6^ TCID_50_ DEV-vectored vaccine provided 100 % protection against duck viral enteritis in ducks, and the hemagglutination inhibition (HI) antibody titer of AIV H5N1 with a peak of 8.2 log_2_ was detected in 3-week-old layer chickens. In contrast, only very weak HI titers were observed in ducks immunized with 10^7^ TCID_50_ DEV-vectored vaccine. A mortality rate of 60 % (6/10) was observed in 1-week-old specific pathogen free chickens inoculated with 10^6^ TCID_50_ DEV-vectored vaccine.

**Conclusions:**

We demonstrate the following in this study. (i) The constructed BAC is a whole genome clone of DEV^C-KCE^. (ii) The insertion of an HA expression cassette sequence into the noncoding area between UL55 and LORF11 of DEV^C-KCE^ affects neither the growth kinetics of the virus nor its protection against DEV. (iii) DEV-H5(UL55) can generate a strong humoral immune response in 3-week-old chickens, despite the virulence of this virus observed in 1-week-old chickens. (iv) DEV-H5(UL55) induces a weak HI titer in ducks. An increase in the HI titers induced by DEV-vectored HA(H5) will be required prior to its wide application.

## Background

Duck enteritis virus (DEV), also known as duck plague, is an important pathogen of ducks, which causes an acute infectious disease with a very high mortality, reaching up to 100 % in birds such as ducks, geese, and wild waterfowls in the order Anseriformes [1, 2]. DEV cases have been reported in many countries, including the United states and China [3, 4]. DEV, also called anatid herpesvirus 1, is a member of the *Mardivirus* genus in the *Alphaherpesvirinae* subfamily of the *Herpesviridae* family in the order *Herpesvirales.* The whole genomes of attenuated and virulent strains of DEV have been sequenced and annotated, which are approximately 158 kbp in length and contain 78 predicted open reading frames (ORFs) of putative proteins [5, 6].

Bacterial artificial chromosomes (BACs) of a few herpesviruses have been previously established [7–9]. Several mutant viruses have been generated by the BAC mutagenesis protocol to study their pathology or their potency as vectors [10–14]. The first DEV BAC was constructed based on a virulent strain (V2085) isolated from the dead ducks in an outbreak in Germany [2, 9]. A DEV-vectored vaccine harboring the hemagglutinin (HA) of the highly pathogenic avian influenza virus (AIV) subtype H5N1 was generated based on this BAC, and robust expression of HA was confirmed in the infected cells [9]. However, the safety of this vaccine remains questionable owing to its development from a virulent parental strain. Nevertheless, this proof-of-principle study clearly demonstrated the potency of a DEV-vectored vaccine expressing AIV HA as a candidate vaccine against AIV.

The AIV H5N1 has attracted considerable attention worldwide owing to its high morbidity and mortality and its potential to mutate into a highly pathogenic form [15–19]. Birds are the main hosts of AIV, but human infections of some strains have been reported. Migratory birds are suspected to play an important role in the transmission of AIV and have been related to several AI outbreaks [20–22]. As the main reservoir of AIV H5N1, ducks may serve as a constant source of viral transmission to chickens and other poultry [23]. Therefore, effective control of AIV H5N1 infection in ducks is critical for AI control in poultry and the prevention of human infections.

Live virus-vectored vaccines based on herpesviruses have been studied for decades, and their ability to induce both robust cellular and humoral immunity has been documented [24–27]. Furthermore, several herpesvirus-vectored vaccines have been licensed and are widely used in some countries [28, 29]. In addition to an early study on the DEV V2085 strain-vectored HA (H5N1) [9], another DEV-vectored H5 vaccine (rDEV-us78HA) has been constructed with the cosmid system using four overlapping DNAs of the DEV genome, which provided strong protection against both duck plague and highly pathogenic AIV H5N1 despite eliciting a weak hemagglutination inhibition (HI) titer [30, 31]. In this study, we generated an infectious clone of DEV vaccine strain C-KCE (DEV^C-KCE^) and constructed a DEV-vectored HA (AIV H5N1) vaccine based on the BAC through the *En Passant* method [32]. This DEV-vectored vaccine was constructed by inserting a synthesized HA gene with consensus sequences from the most recently updated AIV H5N1 strains into the noncoding area between UL55 and LORF11. The stability and safety of the DEV-vectored vaccine and its immunogenicity against duck plague and AIV H5N1 were studied in both ducks and chickens.

## Results and discussion

### Generation of recombinant DEV attenuated strain harboring mini-F plasmid sequences

**Fig. 1:**
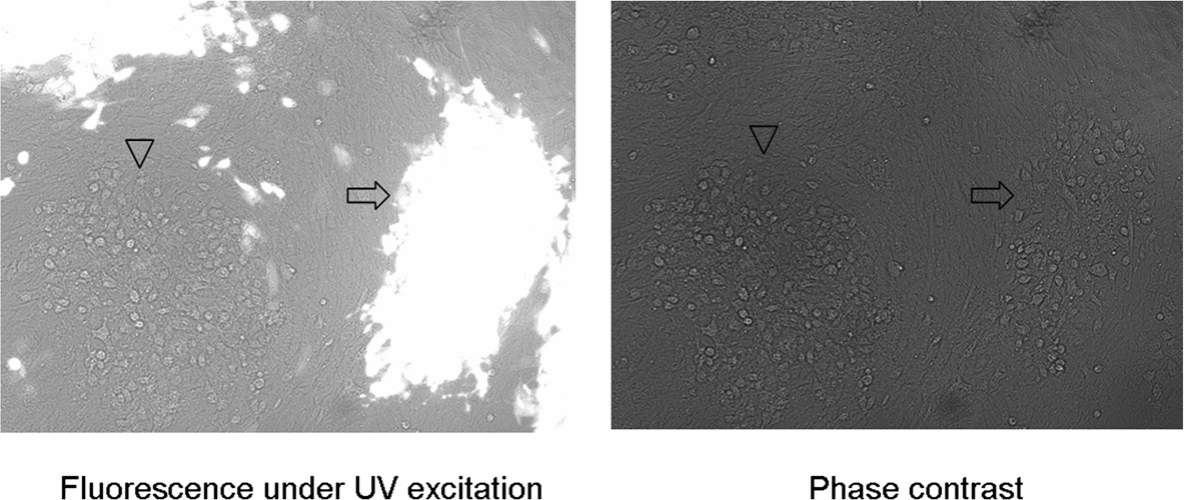
Plaques of DEV^C-KCE^-mini-F and the parental DEV^C-KCE^ virus. Images of DEV^C-KCE^-mini-F and DEV^C-KCE^ plaques under UV excitation (*left*) and phase contrast (*right*) are shown. Arrows show a plaque formed by DEV^C-KCE^-mini-F and arrowheads show a white plaque of parental DEV^C-KCE^ virus. Each panel represents a view of 200 × 200 μm in size

The mini-F sequences were inserted into the DEV^C-KCE^ genome firstly. Three days after the co-transfection of DNA from DEV^C-KCE^ and the BAC transfer vector plasmid pDEVgc-pHA2, a recombinant DEV^C-KCE^ harboring mini-F plasmid sequences was successfully generated as indicated by its production of green fluorescence under UV light (488 nm; Fig. 1). Subsequently, a homogeneous population of mini-F recombinant DEV^C-KCE^ was obtained after three rounds of picking and replating on chicken embryo fibroblasts (CEFs), and named DEV^C-KCE^-miniF.

### Generation of an infectious clone of pDEV^C-KCE^

**Fig. 2:**
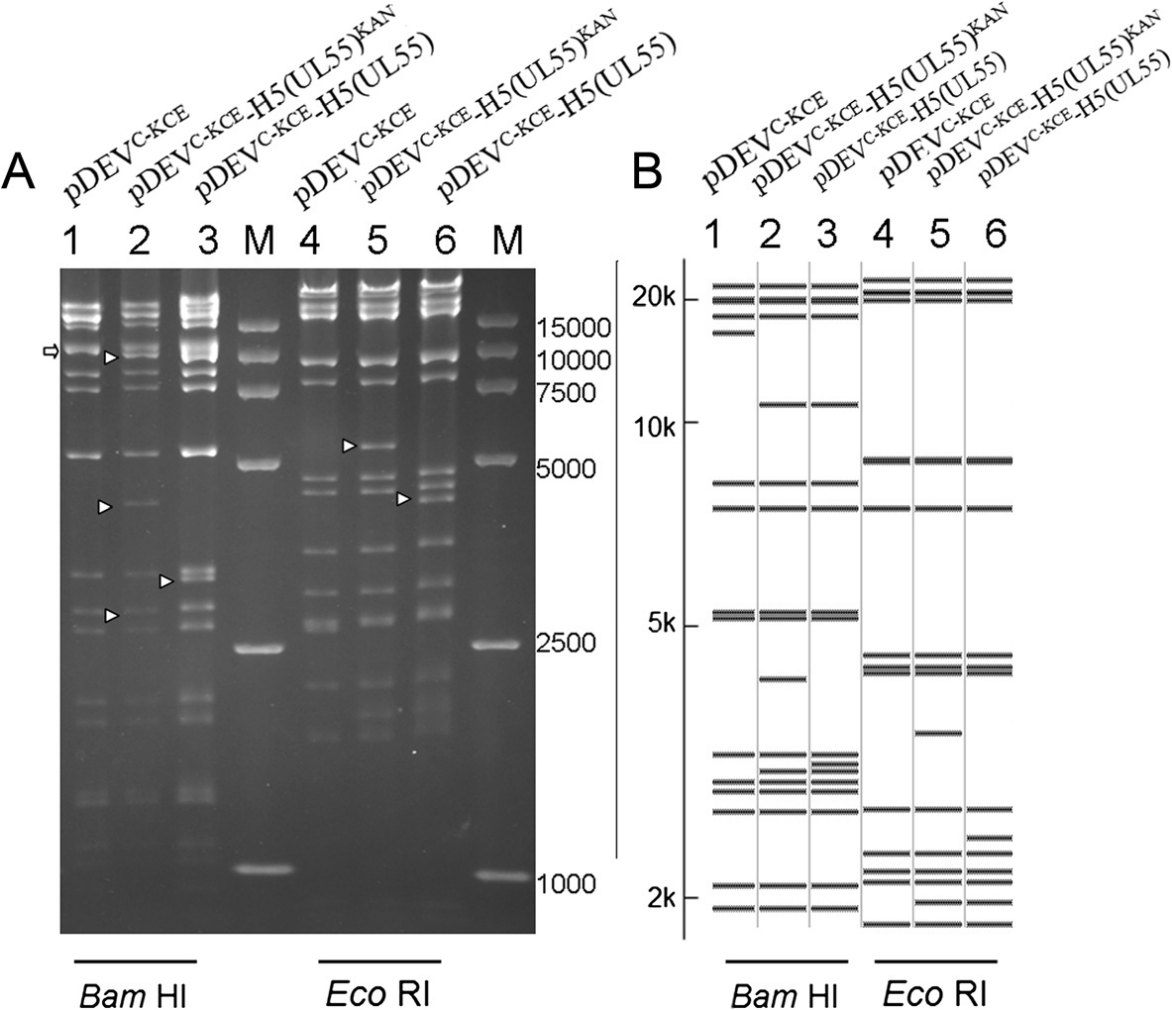
RFLP of DEV^C-KCE^ BAC clone Sa, of pDEV^C-KCE^-H5(UL55)^KAN^, and of pDEV^C-KCE^-H5(UL55). **a** DNA from DEV^C-KCE^ BAC clone Sa (lanes 1 and 4) and recombinant BACs of pDEV^C-KCE^-H5(UL55)^KAN^ (lanes 2 and 5) and pDEV^C-KCE^-H5(UL55) (lanes 3 and 6) were prepared by mini-prep and digested with *Bam*H I (lanes 1–3) or *Eco*R I (lanes 4–6). The digests were separated by 0.8 % agarose gel electrophoresis for 16 h under 40v. Arrowheads highlight differences between the lanes. **b** Predicted RFLP patterns of pDEV^C-KCE^-H5(UL55)^KAN^ and pDEV^C-KCE^-H5(UL55) compared with BAC clone Sa. Size markers range from 2 to 20 kb. Lanes 1–3 show predicted *Bam*H I digestion patterns of Sa, pDEV^C-KCE^-H5(UL55)^KAN^, and pDEV^C-KCE^-H5(UL55), respectively. Lanes 4–6 show predicted *Eco*R I digestion patterns of Sa, pDEV^C-KCE^-H5(UL55)^KAN^, and pDEV^C-KCE^-H5(UL55), respectively. Predictions of these digestions were performed using the DEV (VAC) whole genome sequences as a reference (GenBank ID:EU082088.2). M: DL 15,000 DNA Marker (Takara)

Next, we generated an infections clone of pDEV^C-KCE^. A total of seven colonies with chloramphenicol resistance were obtained after electroporation of DEV^C-KCE^-mini-F DNA into *Escherichia coli* DH10B competent cells, one of which, termed pDEV^C-KCE^, was selected for further restriction fragment length polymorphism (RFLP) analysis. Enzymatic digestion of pDEV^C-KCE^ with *Bam*H I and *Eco*R I showed the expected patterns with slight differences (Fig. 2), which might be caused by the differences between the sequences of DEV^C-KCE^ and the reference genome DEV(VAC) (GenBank ID:EU082088.2). Then, the pDEV^C-KCE^ DNA from the Midi-prep was electroporated into *E. coli* GS1783 competent cells to generate the infectious clone Sa for further construction of mutants through the *En Passant* method. RFLP patterns of Sa with *Bam*H I and *Eco*R I were exactly the same as that of pDEV^C-KCE^.

### Generation of pDEV^C-KCE^-H5(UL55)

**Fig. 3:**
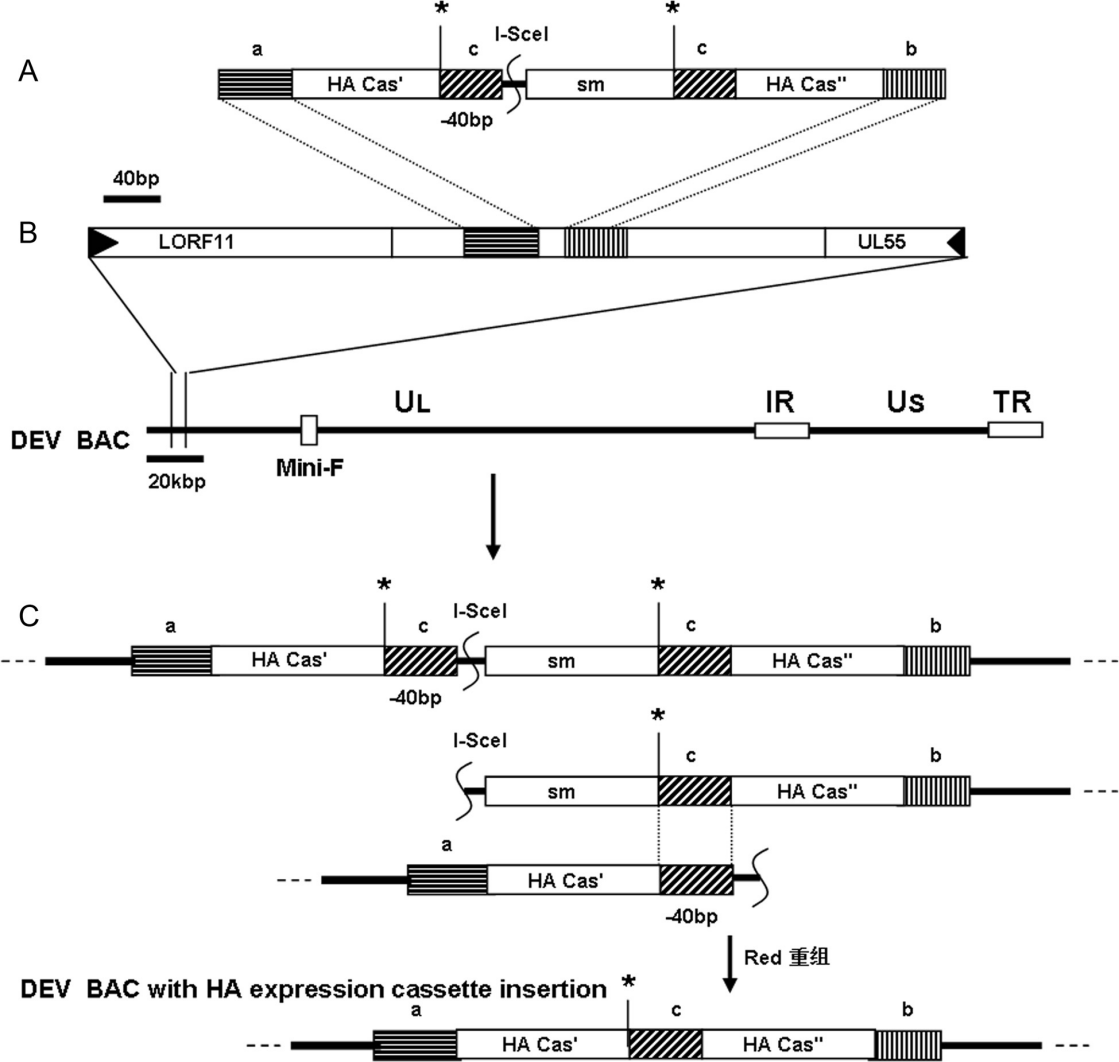
Construction of recombinant pDEV^C-KCE^-H5(UL55). **a** A kanamycin resistance gene (sm) with a 40 bp homologous sequence (c) was inserted into the *Sac* I restriction site (*) in the HA expression cassette, which was divided into two parts (HA Cas’ and HA Cas”). **b** The HA expression cassette with the selective marker (sm) was inserted into the noncoding area between LORF11 and UL55 through the first recombination to generate a recombinant BAC clone (pDEV^C-KCE^-H5(UL55)^KAN^) with both chloramphenicol and kanamycin resistance. **c** The second recombination was performed to delete the kanamycin resistance gene and generate the final recombinant pDEV^C-KCE^-H5(UL55)-vectored HA clone. Rectangles with the same type of shading indicate identical sequences. Scales in bp or kbp are provided

Following the successful generation of the DEV^C-KCE^ clone Sa, this clone was used in the *En Passant* method to generate a recombinant BAC clone (pDEV^C-KCE^-H5(UL55)^KAN^) with both chloramphenicol and kanamycin resistance. The HA expression cassette harboring a kanamycin resistance gene was inserted through the first recombination with the DEV^C-KCE^ BAC clone Sa. The kanamycin resistance gene was successfully deleted from the HA expression cassette through a second recombination to generate the DEV recombinant BAC, named pDEV^C-KCE^-H5(UL55), which harbored the HA expression cassette in the noncoding area between UL55 and LORF11 (Fig. 3). RFLP analysis of the *Bam*H I digestion of these constructs showed that DEV^C-KCE^ BAC clone Sa lacked a band around 13 kbp (lane 1, Fig. 2a) while three bands of approximately 10 kbp, 4.8 kbp, and 2.7 kbp were observed in pDEV^C-KCE^-H5(UL55)^KAN^ (lane 2, Fig. 2a). Moreover, pDEV^C-KCE^-H5(UL55) (lane 3, Fig. 2a) lacked a 4.8 kbp band, and instead had a band of approximately 3.8 kbp. These results are consistent with the expected patterns (Fig. 2b, lanes 1, 2, and 3), suggesting the successful insertion of the HA expression cassette and the deletion of kanamycin resistance gene. After digestion with *Eco*R I, a band of approximately 5.5 kbp was observed in pDEV^C-KCE^-H5(UL55)^KAN^ (lane 5, Fig. 2a) that was not observed in DEV^C-KCE^ BAC clone Sa or in pDEV^C-KCE^-H5(UL55) (lane 6, Fig. 2a). However, pDEV^C-KCE^-H5(UL55) (lane 6, Fig. 2a) had a unique band of approximately 4.5 kbp. These results support the successful insertion of a HA cassette and the deletion of a kanamycin resistance gene, even though they are slightly different from the expected pattern (Fig. 2b). These differences might be due to the different origins of DEV^C-KCE^ and the reference strain DEV(VAC), even though they both claimed to be the Chinese commercial vaccine strain. The correct sequences of the inserted HA cassette were confirmed by sequencing.

### Rescue of recombinant DEV from BAC and generation of DEV mutants

After co-transfection of DNA from pDEV^C-KCE^ and a polymerase chain reaction (PCR) fragment that was amplified using DEV^C-KCE^ DNA as template and a pair of primers (DEV-HOMO1-for and DEV HOMO2-rev) [9], nonfluorescent plaques were observed under UV light (488 nm). A homogeneous population of viruses was isolated by three rounds of picking and plating purification. The expected 3923 bp band was amplified by PCR with primers (DEV gC flanking F and DEV gC flanking R) and sequencing results showed that the complete glycoprotein C (gC) gene was recovered from the same place as in the parental virus. The resulting gC-recovered DEV virus was termed DEV^C-KCE^gC^R^.

The DEV^C-KCE^-harboring HA, termed DEV-H5(UL55), was successfully generated with gC recovered in a similar way as for DEV^C-KCE^gC^R^ using pDEV^C-KCE^-H5(UL55) DNA. The DEV-H5(UL55) HA expression cassette was amplified by PCR with primers (DEV ins H5 casse UL55 F and DEV ins H5 casse UL55 R), and its correct insertion was confirmed by sequencing with the 20 specific sequencing primers (Table 1).

**Table 1:** Primers for PCR and sequencing

Primer	Sequence
Kan ins’ H5(HA) F	5′-*TTA*gagctcCTCGCTGCAGGCGGCCGCTCTAGAACTCGTCGATCGCAGCGGGATGACGACGATAAGTAGGGATAAC-3′ ^a^
Kan ins’ H5(HA) R	5′-*CGC*gagctcGGGTAATGCCAGTGTTACAACCA-3′ ^a^
DEV ins H5 casse UL55 F	5′-CGACGGACTGCCAGTGAACGCTGAACAAGCTAGGACAATTCTAGTGGATCCCCCAACTCC-3′
DEV ins H5 casse UL55 R	5′-AAGTAAAGACCCAAGCTACTAACAGGGTATTTGGGTAATATTGTCGACTCTAGAGGATCCG-3′
DEV gC flanking F	5′-TTCGCCGTATTTACCAAATG-3′
DEV gC flanking R	5′-TGATTCCTTTTGTTCGGATA-3′
DEV H5(UL55) casse seq F1	5′-CTAGTGGATCCCCCAACTCC-3′
DEV H5(UL55) casse seq F2	5′-GTACATTGGGTCAATGGGAG-3′
DEV H5(UL55) casse seq F3	5′-AAGTACACTGCGTCAATAGG-3′
DEV H5(UL55) casse seq F4	5′-ACTTTCCAATGGGTTTTGCC-3′
DEV H5(UL55) casse seq F5	5′-GCTGATTAATGGGAAAGTAC-3′
DEV H5(UL55) casse seq F6	5′-CGATCATATTTGCATTGGTT-3′
DEV H5(UL55) casse seq F7	5′-TTGAAACACCTATTGAGCAG-3′
DEV H5(UL55) casse seq F8	5′-GGACATCAACACTAAACCAG-3′
DEV H5(UL55) casse seq F9	5′-GAATGTCCCAAATATGTGAA-3′
DEV H5(UL55) casse seq F10	5′-CTTAGAGAGGAGAATAGAGAAT-3′
DEV H5(UL55) casse seq R1	5′-TTGTCGACTCTAGAGGATCC-3′
DEV H5(UL55) casse seq R2	5′-CATCCATAAAGATAGACCAG-3′
DEV H5(UL55) casse seq R3	5′-ATGGAAGTCTAGAGTTCTCTC-3′
DEV H5(UL55) casse seq R4	5′-TAAAACCTGCTATAGCTCCA-3′
DEV H5(UL55) casse seq R5	5′-CGGTTTTAAAATTGTCCAGA-3′
DEV H5(UL55) casse seq R6	5′-TGGACATGCTGCACTCACCC-3′
DEV H5(UL55) casse seq R7	5′-TTTCCAGTATGTCTTGGGCA-3′
DEV H5(UL55) casse seq R8	5′-ATATGGAATTTCCAGGGGAA-3′
DEV H5(UL55) casse seq R9	5′-CCCAATGGAAAGTCCCTATT-3′
DEV H5(UL55) casse seq R10	5′-TCAGTGTACTTGGCTCCAAT-3′

^a^ restriction enzyme sites added to primers are in bold lower case letters, and sequences in italics indicate additional bases that are not present in the original DEV genome

### Stability and growth kinetics of mutant or gC-recovered DEV

The DEV mutant with HA insertion, DEV-H5(UL55), was replicated serially on CEFs for 20 passages (F20). To test the stability of this virus, a fragment of approximately 3360–3390 bp was amplified with F20 virus DNA as a template and a pair of primers (DEV ins H5 casse UL55 F and DEV ins H5 casse UL55 R). The HA expression cassette sequences were confirmed by sequencing, indicating that the inserted HA expression cassette was stable for at least 20 passages.

**Fig. 4:**
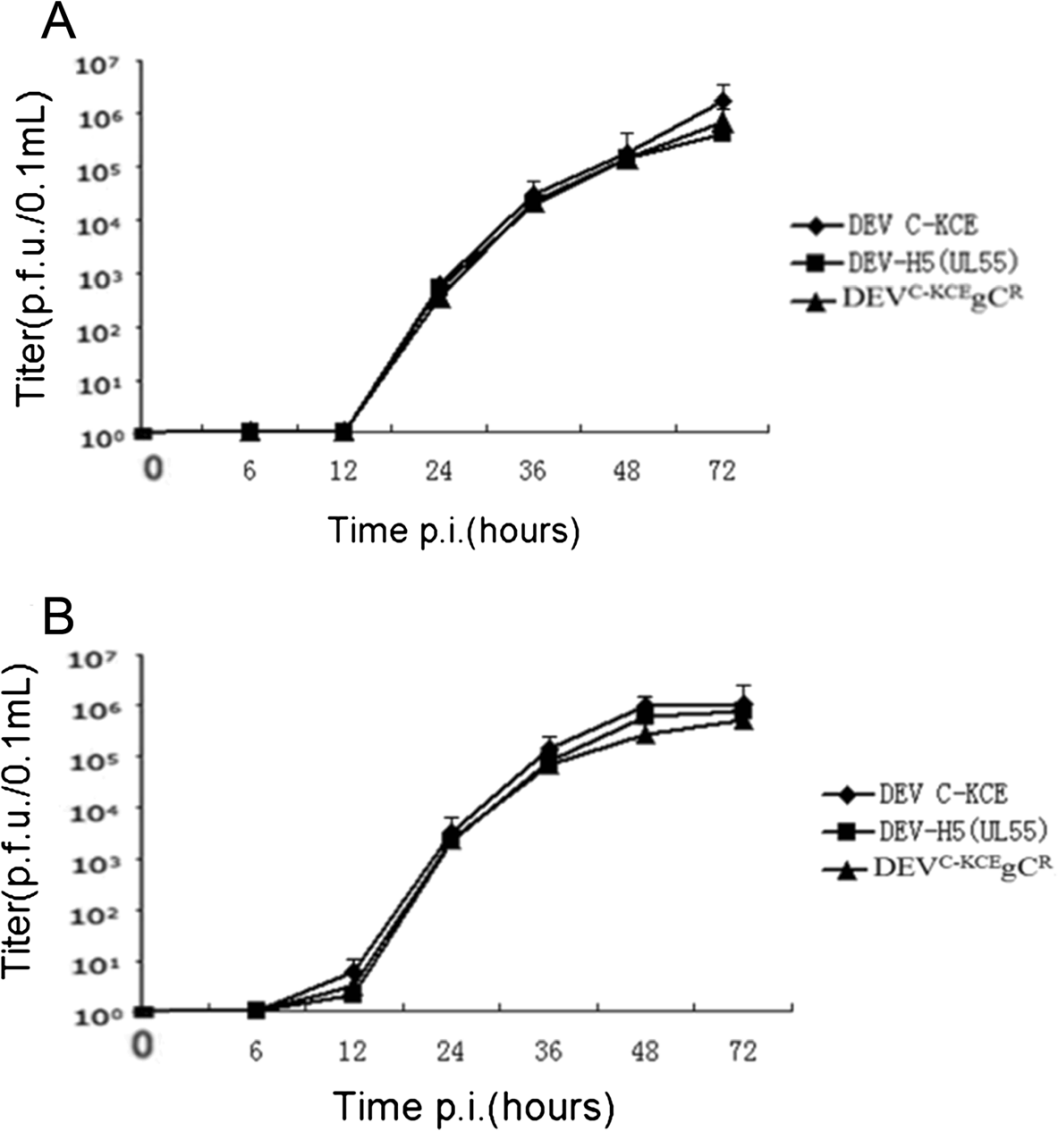
Comparison of DEV^C-KCE^gC^R^, DEV-H5(UL55), and DEV^C-KCE^ multi-step growth kinetics on CEFs. **a** Viral titers of infected-cell supernatants. **b** Viral titers of cell-associated viruses. Titers were determined as the number of plaque forming units in 0.1 ml of sample in three independent tests. Average titers were determined at the indicated time points after infection with an MOI of 0.01. Error bars represent the standard deviations

Multi-step growth kinetics of gC-recovered DEV virus (DEV^C-KCE^gC^R^), made from pDEV^C-KCE^, and of DEV-vectored HA recombinant virus (DEV-H5(UL55)), made from pDEV^C-KCE^-H5(UL55), were compared with the parental virus (DEV^C-KCE^) in three independent experiments. No virus titers were detected at 12 h post-infection (p.i.) in infected cell supernatants and the titers peaked (around 10^6^ plaque-forming units (pfu)/0.1 ml) at 72 h p.i. for all virus strains. No significant differences were observed between DEV-H5(UL55) and DEV^C-KCE^ (*p* = 0.200 and *p* = 0.125 at 48 h and 72 h p.i., respectively) or between DEV^C-KCE^gC^R^ and DEV^C-KCE^ (*p* = 0.122 and *p* = 0.055 at 48 h and 72 h p.i., respectively) (Fig. 4a).

The titers of viruses released from infected cells were determined after three freeze–thaw cycles. Results revealed a slightly lower titer of the gC revertant viruses (DEV^C-KCE^gC^R^ and DEV-H5(UL55)) compared with that of the parental virus (DEV^C-KCE^). However, these differences were not significant between DEV-H5(UL55) and DEV^C-KCE^ (*p* = 0.094 and *p* = 0.154 at 48 h and 72 h p.i., respectively) or between DEV^C-KCE^gC^R^ and DEV^C-KCE^ (*p* = 0.164 and *p* = 0.322 at 48 h and 72 h p.i., respectively) (Fig. 4b). These results prove that the DEV^C-KCE^ BAC clone is a whole genome clone of the parental virus and the genetic manipulation of DEV-vectored HA during the *En Passant* recombination did not affect the integrity of the genome. The results also show that the insertion of a foreign gene expression cassette between the noncoding area of UL55 and LORF11 did not affect either the growth kinetics of the virus or the stability of the inserted sequences. These findings support the development of the constructed DEV-H5(UL55) as a live vector vaccine candidate.

### Expression of AIV HA by recombinant DEV-H5(UL55)

**Fig. 5:**
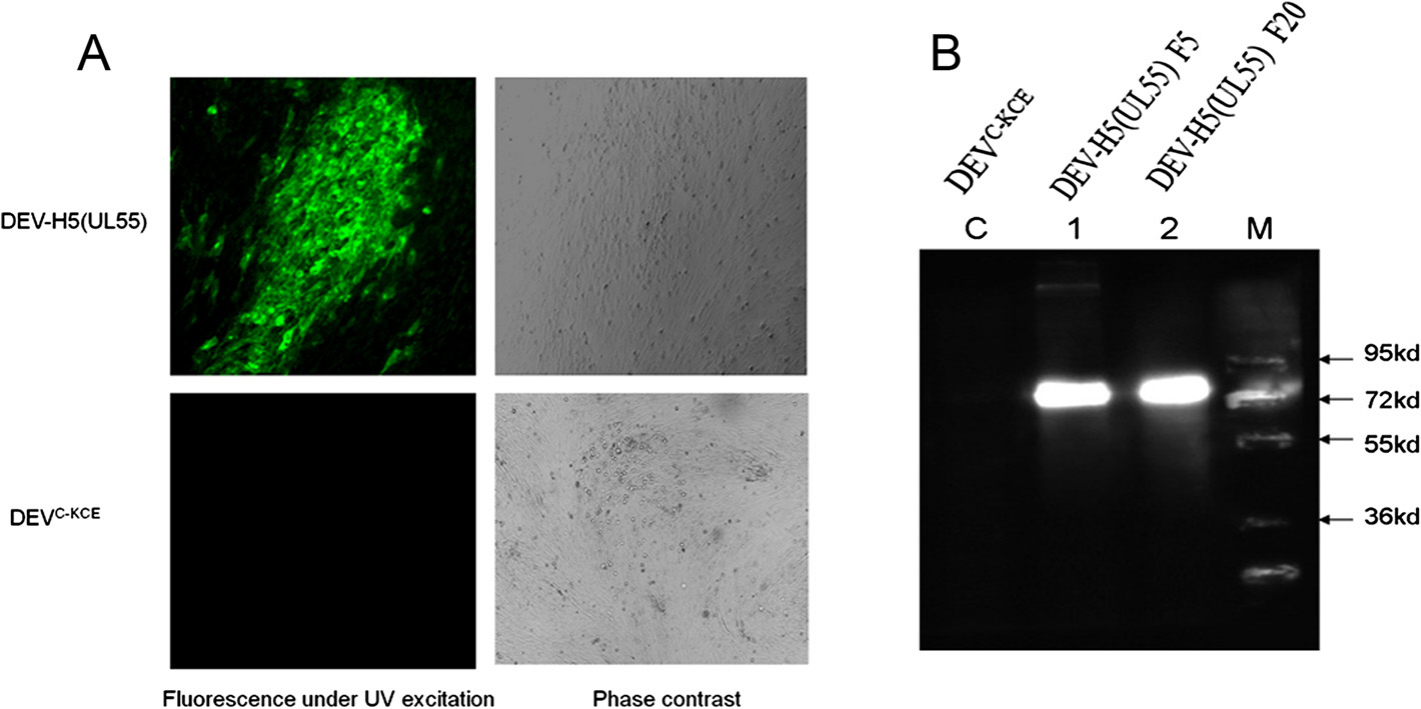
IIF and western blotting analysis of DEV-H5(UL55) HA expression. **a** IIF was performed with a mixture of AIV H5N1 monoclonal antibodies. Images of DEV^C-KCE^ (lower) and DEV-H5(UL55) F20 (upper) are shown under UV excitation (left) and in phase contrast (right). Each individual panel represents a view of 200 × 200 μm in size. **b** Western blotting of HA expression of DEV-H5(UL55). Lane C: lysates of CEFs infected with DEV^C-KCE^. Lanes 1 and 2: lysates of CEFs infected with DEV-H5(UL55) F5 and F20, respectively. Proteins were separated by SDS-10 % polyacrylamide gel electrophoresis and transferred to nitrocellulose membranes (Merck). A mixture of H5 monoclonal antibodies (Genescript) were used as the primary antibodies and a 1:10,000 dilution of goat-anti-mouse IgG (ABcom) was used as the secondary antibody. Detection was performed with enhanced chemiluminescence (Sigma-Aldrich). Lane M: DNA marker (PageRuler^TM^ Plus, Fermentas)

In indirect immunofluorescence (IIF) assays, plaques of DEV-H5(UL55) F20 reacted strongly with the mixture of monoclonal antibodies against AIV H5N1, generating intense signals under UV light (488 nm), plaques of the control DEV^C-KCE^ displayed no signals under the same conditions (Fig. 5a). In western blotting, although proteins with molecular masses of approximately 80 kiloDaltons (KDa) were observed in lysates of CEFs infected with either DEV-H5(UL55) that had been passaged five times (F5) or 20 times (F20), no protein bands were detected in CEFs infected with DEV^C-KCE^ (Fig. 5b). The sizes of the detected bands from DEV-H5(UL55) F5 and F20 (80 KDa) were slightly bigger than the estimated size (68 KDa) based on the primary amino acid sequences of synthesized HA. A similar phenomenon was reported in a previous study on DEV v2085_H5 HA expression and was shown to be related to the HA protein N-linked glycans [9]. In this study, we confirmed the robust expression of HA in the recombinant DEV-H5(UL55) after 20 passages.

### Safety and immunogenicity of DEV-H5(UL55) in ducks

**Fig. 6:**
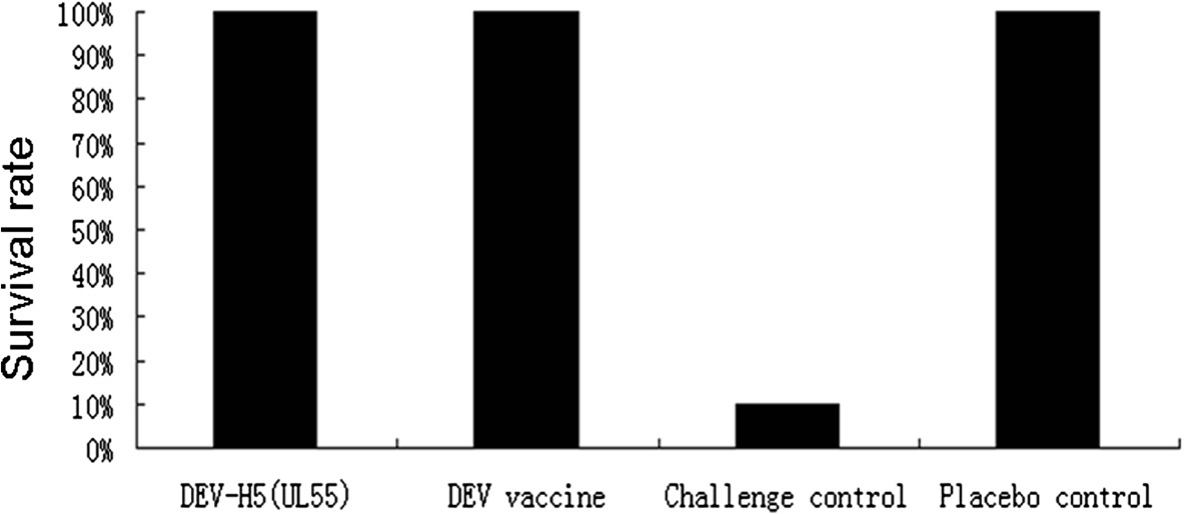
Protection efficiency of DEV-H5(UL55) against duck plague. A total of 40 commercial ducks were randomly divided into four groups. Ducks in groups A-DP(5D) (Column DEV-H5(UL55)) and B-DP(5D) (Column DEV vaccine) were inoculated intramuscularly with 1 × 10^6^ TCID_50_ DEV-H5(UL55) virus and DEV vaccine(NJTB), respectively. Groups C-DP(5D) (challenge control) and D-DP(5D) (placebo control) were inoculated intramuscularly with 0.2 ml of PBS as controls. All of the ducks in groups A-DP(5D), B-DP(5D), and C-DP(5D) were challenged with virulent DEV at a dose of 100 LD_50_ and monitored daily for 14 days. The survival rates of these four groups at the end of the 14 days are shown here

To test the safety of DEV-H5(UL55) and its efficacy against virulent DEV, ducks in groups A-DP(5D) and B-DP(5D) were inoculated intramuscularly with 1 × 10^6^ TCID_50_ DEV-H5(UL55) virus and DEV vaccine (NJTB), respectively, and ducks in groups C-DP(5D) (challenge control) and D-DP(5D) (placebo control) were inoculated intramuscularly with phosphate-buffered saline (PBS) as controls. All of the ducks in groups A-DP(5D), B-DP(5D), and C-DP(5D) were challenged with 100 LD_50_ virulent DEV. No clinical signs were observed in any of the ducks in group A-DP(5D). From day 3 post-challenge (d.p.c.), ducks in group C-DP(5D) began to show symptoms of duck plague, including elevation of feathers, lethargy, loss of appetite, greenish or water-like diarrhea, and tremors of necks. Dead ducks were observed from 4 d.p.c. At the end of the test, 9/10 of the ducks in group C-DP(5D) died from duck plague, whereas all the ducks in groups A-DP(5D), B-DP(5D), and D-DP(5D) were healthy throughout the observation period (Fig. 6). These results suggest that a single injection of the virus rescued from the constructed BAC provides the same strong immunity against duck plague as vaccination with its parental virus. Moreover, these results further confirm that the constructed DEV BAC in this study contains the whole genome of the parental virus and that the insertion of the synthesized HA expression cassette into the DEV BAC did not affect its immunity against duck plague. They also show that the resulting recombinant DEV-H5(UL55) is safe in ducks.

**Fig. 7:**
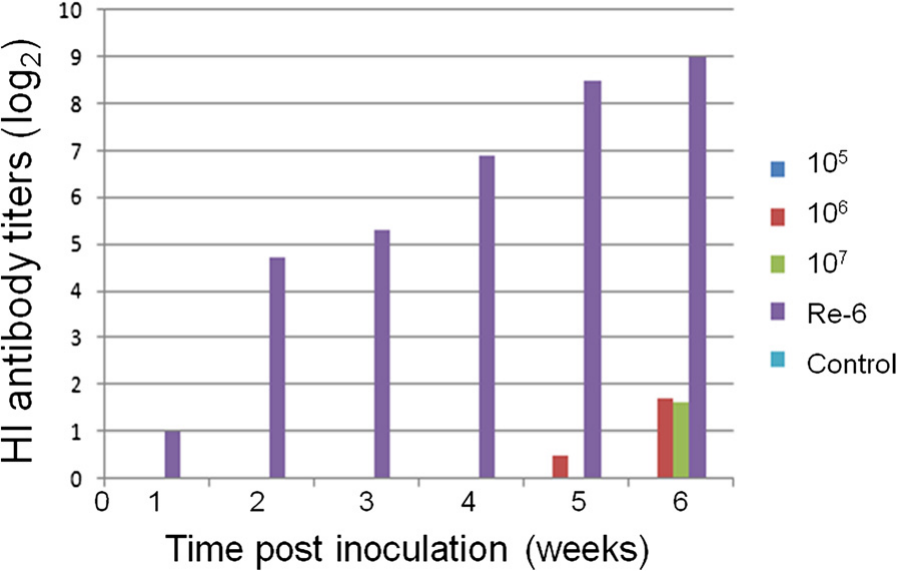
HI antibody titers against AIV H5N1 in commercial ducks vaccinated with DEV-H5(UL55). A total of 50 2-week-old commercial ducks were randomly divided into five groups of A-AI(2D), B-AI(2D), C-AI(2D), D-AI(2D), and E-AI(2D). Birds in groups A-AI(2D), B-AI(2D), and C-AI(2D) were vaccinated intramuscularly with DEV-H5(UL55) at a dose of 1 × 10^7^ TCID_50_ (Lane: 10^7^), 1 × 10^6^ TCID_50_ (Lane: 10^6^) and 1 × 10^5^ TCID_50_ (Lane: 10^5^), respectively. Birds in group D-AI(2D) were inoculated subcutaneously with a dose of 0.5 ml and 1 ml of inactivated AIV H5N1 Re-6 vaccine (QYH) at 2 w and 5 w, respectively, according to the manufacturer’s instructions (Lane: Re-6). Birds in the control group E-AI(2D) were inoculated with 0.2 ml of PBS (Lane: Control). Serum samples of all birds were collected prior to inoculation and at 1, 2, 3, 4, 5, and 6 weeks after vaccination to measure the HI titers, which are shown here

To further test the safety of DEV-H5(UL55) and to test its immunogenicity against AI, ducks in groups A-AI(2D), B-AI(2D), and C-AI(2D) were vaccinated intramuscularly with DEV-H5(UL55) at a dose of 1 × 10^7^, 1 × 10^6^, and 1 × 10^5^ TCID_50_, respectively. Birds in group D-AI(2D) were inoculated twice subcutaneously with inactivated AIV H5N1 Re-6 vaccine (QYH). Birds in the control group E-AI(2D) were inoculated with PBS. No clinical signs were observed in ducks from any of the groups, and no HI antibodies were detected in any of the ducks in group E-AI(2D). No HI antibodies were detected in any of the ducks in groups A-AI(2D), B-AI(2D), and C-AI(2D) until 4 weeks post-inoculation. At as late as 5 weeks post-inoculation, a HI titer of 5 log_2_ was detected in only one of the 10 ducks in group B-AI(2D). Although HI titers of 2–7 log_2_ were detected in four of the 10 ducks in group B-AI(2D) and three of the 10 ducks in group C-AI(2D) at 6 weeks post-inoculation, no HI antibodies were observed in any of the remaining ducks in these two groups nor was any detected in the ducks in group A-AI(2D). Meanwhile, in group D-AI(2D), an average HI titer of as high as 8.5 log_2_ was detected at 1 week after the boost vaccination with the inactivated vaccine (Fig. 7). These results indicate that DEV-H5(UL55) stimulated only a weak humoral immunity against HA(H5N1) in commercial ducks.

This phenomenon was also observed in a previous study on a DEV-vectored HA(H5N1) vaccine (rDEV-us78HA), which was generated with the insertion of a HA expression cassette, composed of the SV40 promoter and the HA from H5N1 AIV AH/1, into the noncoding area between ORF US7 and US8. An HI titer of as low as 3 ~ 4 log_2_ was detected in specific pathogen-free (SPF) ducks inoculated with two doses of rDEV-us78HA vaccine separated by a 3-week interval at 4 weeks after inoculation; however, 100 % protection was observed in a challenge test with a highly pathogenic avian influenza H5N1 subtype virulent strain (HB/49) [30]. In our study, DEV-H5(UL55) was constructed with the insertion of a pMCMV promoter and a consensus HA sequence into the noncoding area between LORF11 and UL55. Although it was demonstrated that the protective potency of rDEV-us78HA was not related to the HI titer elicited by inoculation of the vaccine [30], the low HI titer will seriously hinder the wide application of the DEV-vectored H5N1 AIV vaccine because the surveillance system for the outcome of vaccination strategy depends largely on the examination of HI titer. Further studies will be needed to improve this DEV-vectored vaccine in order to elicit much higher HI titers in ducks in advance of intensive challenge tests with H5N1 AIV.

### Safety and immunogenicity of DEV-H5(UL55) for chickens

**Fig. 8:**
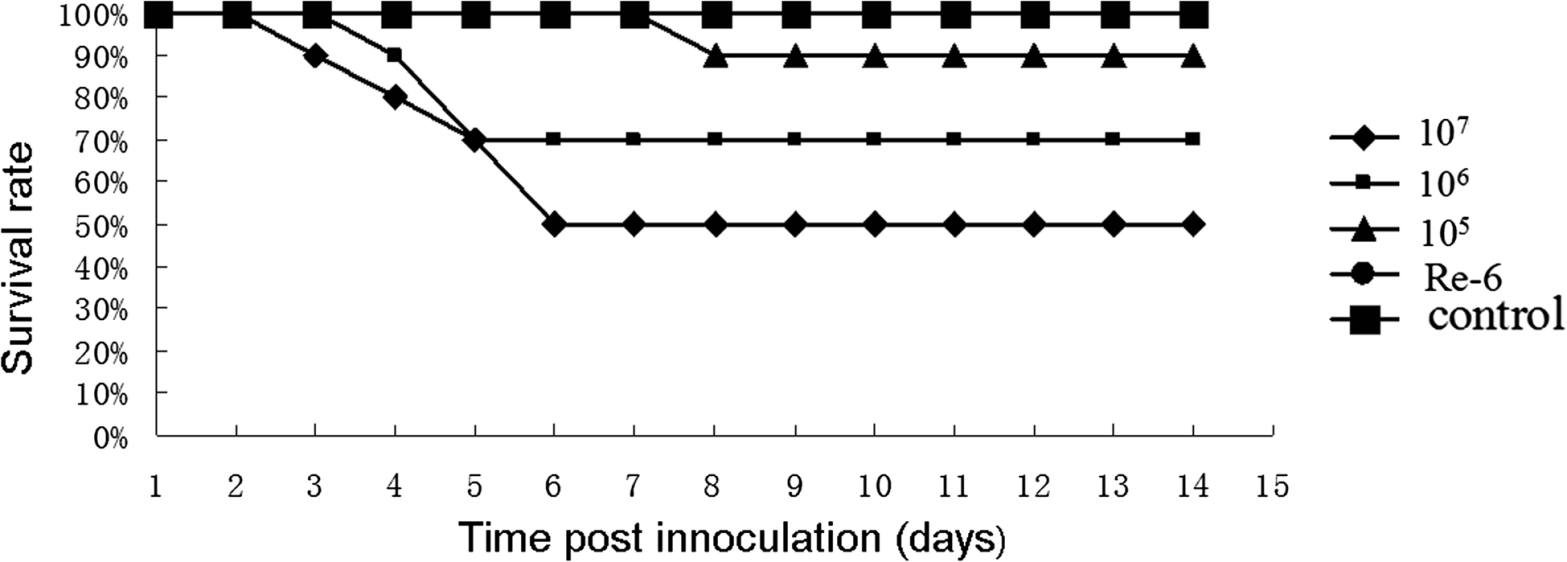
Safety of DEV-H5(UL55) for chickens. A total of 50 1-week-old SPF chickens were randomly divided into five groups of A-AI(1C), B-AI(1C), C-AI(1C), D-AI(1C), and E-AI(1C). In groups A-AI(1C), B-AI(1C), and C-AI(1C), chickens were vaccinated intramuscularly with DEV-H5(UL55) at a dose of 1 × 10^7^ TCID_50_ (Line 10^7^), 1 × 10^6^ TCID_50_ (Line: 10^6^), and 1 × 10^5^ TCID_50_ (Line: 10^5^), respectively. Chickens in group D-AI(1C) were inoculated subcutaneously with a dose of 0.3 ml of inactivated AIV H5N1 Re-6 vaccine (QYH) (Line: Re-6). Chickens in the control group E-AI(1C) were inoculated with 0.2 ml PBS (Line: Control). All birds were observed for clinical signs over the 14 days after inoculation. The survival rates of these five groups over the 14 days are shown here

To test the safety of DEV-H5(UL55) and its efficacy against virulent DEV, chickens in groups A-AI(1C), B-AI(1C), and C-AI(1C) were vaccinated intramuscularly with DEV-H5(UL55) at a dose of 1 × 10^7^, 1 × 10^6^, and 1 × 10^5^ TCID_50_, respectively. Chickens in group D-AI(1C) were inoculated subcutaneously with inactivated AIV H5N1 Re-6 vaccine (QYH), and chickens in the control group E-AI(1C) were inoculated with PBS. All 1-week-old SPF chickens were healthy before inoculation, and no HI antibodies were detected in any of the serum samples collected prior to inoculation or in control group E-AI(1C) throughout the experiment. Starting from 3 days post-immunization (d.p.i.), all of the chickens in groups A-AI(1C), B-AI(1C), and C-AI(1C) showed clinical signs of duck plague, such as greenish diarrhea, slow reaction, lethargy, inability to stand, and loss of appetite. A total of six, three, and one birds died between 3 and 10 d.p.i. in groups A-AI(1C), B-AI(1C), and C-AI(1C), respectively (Fig. 8). Lesions of dead birds exhibited typical xanthochromia in subcutaneous fat or gelatinous materials, hemorrhage in proventriculus and pectoralis muscles, necrotic foci in liver, and renomegaly. The presence of DEV gC in the liver samples of dead birds was confirmed by PCR with a pair of specific primers (DEV gC flanking F and DEV gC flanking R). Birds in groups D-AI(1C) and E-AI(1C) were healthy throughout the experiment (Fig. 8). Results from the HI antibody test showed that the birds in groups A-AI(1C), B-AI(1C), and C-AI(1C) developed immune responses no later than 2 weeks post-inoculation with the DEV-vectored vaccine, whereas no HI antibodies were detected in the samples from birds in group D-AI(1C). At 3 weeks post-inoculation, the average peak HI titers were 6.6, 5.6, and 5.5 log_2_ in the surviving birds in groups A-AI(1C), B-AI(1C), and C-AI(1C), respectively. Meanwhile, a HI titer of 7.6 log_2_ was detected in group D-AI(1C).

**Fig. 9:**
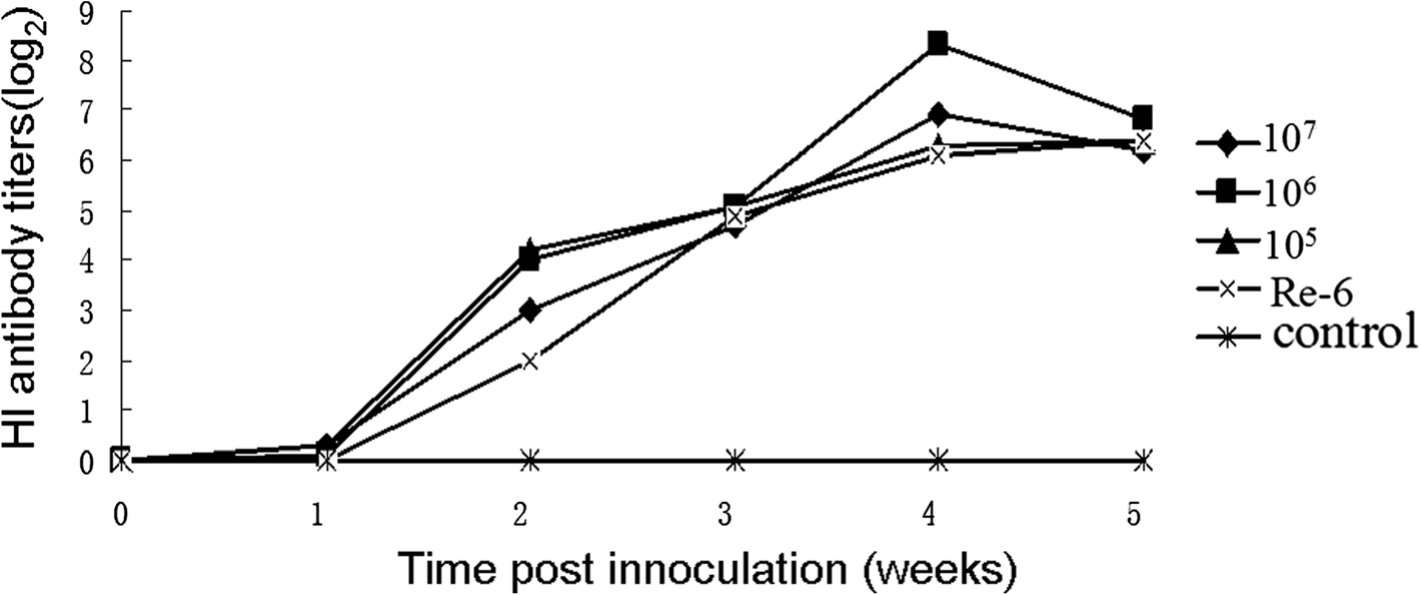
HI antibody titers against AIV H5N1 in chickens vaccinated with DEV-H5(UL55). A total of 50 3-week-old commercial chickens were randomly divided into five groups of A-AI(3C), B-AI(3C), C-AI(3C), D-AI(3C), and E-AI(3C). In groups A-AI(3C), B-AI(3C), and C-AI(3C), chickens were vaccinated intramuscularly with DEV-H5(UL55) at a dose of 1 × 10^7^ TCID_50_ (Line: 10^7^), 1 × 10^6^ TCID_50_ (Line: 10^6^), and 1 × 10^5^ TCID_50_ (Line: 10^5^), respectively. Chickens in group D-AI(3C) were inoculated subcutaneously with a dose of 0.3 ml of inactivated AIV H5N1 Re-6 vaccine (QYH) according to the manufacturer’s instructions (Line: Re-6). Chickens in the control group E-AI(3C) were inoculated with 0.2 ml of PBS (Line: Control). Serum samples of all chickens were collected before inoculation and at 1, 2, 3, 4, 5, and 6 weeks after vaccination to measure the HI titers, which are shown here

To further test the safety of DEV-H5(UL55) and to test its immunogenicity against AI, 3-week-old chickens in groups A-AI(3C), B-AI(3C), and C-AI(3C) were vaccinated intramuscularly with DEV-H5(UL55) at a dose of 1 × 10^7^, 1 × 10^6^, and 1 × 10^5^ TCID_50_, respectively. Chickens in group D-AI(3C) were inoculated subcutaneously with inactivated AIV H5N1 Re-6 vaccine (QYH), and chickens in the control group E-AI(3C) were inoculated with PBS. The chickens in all groups were healthy throughout the experiment. At only 1 week post-inoculation, HI antibodies were detected in two or three birds in groups A-AI(3C), B-AI(3C), and C-AI(3C), whereas no HI antibodies were detected in any of the birds in group D-AI(3C). At 4 weeks post-inoculation, HI titers in groups A-AI(3C), B-AI(3C), and C-AI(3C) peaked 6.9, 8.3, and 6.3 log_2_, respectively, which were higher than the average titer of 6.1 log_2_ in the birds in group D-AI(3C) (Fig. 9). These results demonstrate that DEV-H5(UL55) induced efficient immunity (both cellular and humoral immunity) against AIV more quickly compared with the inactivated vaccine. However, this virus showed virulence for young chickens. The parental virus, C-KCE strain, was isolated from liver samples from ducks that died of duck plague and was attenuated through serial passages in chick embryos [5, 30]. This process might increase the virulence of viruses for young chickens. Nevertheless, the robust immunity induced in chickens might be due to the efficient replication of the virus after inoculation.

While the induction of high HI titers in chickens inoculated with DEV-H5(UL55) provides strong evidence for its ability to activate humoral immunity, the high HI titers induced in ducks vaccinated with an inactivated AIV vaccine exclude the possibility that ducks have an inherently low reaction to AIV HA as an antigen. A possible explanation for the extremely weak HI titers in ducks inoculated with DEV-H5(UL55) might be that DEV interferes with duck immunity. It is known that some herpesvirus proteins can interfere with the host immune reaction. This interference is an important mechanism of interaction between viruses, such as Marek’s disease virus and herpes simplex virus, and their hosts during evolution [33, 34]. This phenomenon might also hold true for DEV-vectored vaccines. Furthermore, the vaccination strategy for control of AIV H5N1 in many countries, including China, depends on a reliable detection method to evaluate the efficacy of vaccination. The improved ability of DEV-H5(UL55) to induce a much higher HI titer in ducks is a prerequisite for the wide application of this DEV-vectored vaccine. Future studies might include deletion or modification of suspected immune interference related genes in the DEV-vectored vaccine.

## Conclusions

In this study, an infectious clone of DEV vaccine strain C-KCE was successfully constructed with the insertion of mini-F sequences in lieu of gC. Growth kinetics, RFLP, and animal tests of gC-recovered viruses or BACs were performed to show that this clone consists of the whole C-KCE strain genome. Further, a DEV-vectored vaccine harboring a synthesized HA expression cassette between the ORF of UL55 and LORF11 was efficiently generated through the *En Passant* protocol based on this infectious clone. Additionally, animal tests showed that this DEV-vectored vaccine was safe in ducks and that one dose of the vaccine provided 100 % protection against duck plague. Although one dose of the vaccine induced high HI titers (8.3 log_2_) against AIV H5N1 in 3-week-old commercial layer chickens, it only stimulated weak HI titers in commercial ducks. Moreover, the DEV-vectored vaccine was virulent in young chickens. Future studies that include the deletion or modification of genes associated with immune regulation and virulence will be required prior to any wide application of the DEV-vectored HA(H5) vaccine. Once this vaccine has been modified to induce a higher HI titer, challenge tests to evaluate its ability to protect against AI will be carried out with homogeneous or heterogeneous viruses of AIV H5N1.

## Methods

### Viruses and plasmids

A DEV attenuated strain (C-KCE strain, DEV^C-KCE^), the widely used commercial vaccine strain attenuated by serial passaging in SPF chicken embryonated eggs, was isolated from a batch of commercial vaccine provided by the Nanjing Tech-bank Bio-industry Co., Ltd. (Nanjing, China) and then purified through three rounds of plaque picking. DEV virulent virus was obtained from the China Veterinary Culture Collection Management Center. All DEV strains were propagated on primary or secondary CEFs. Virus stocks were prepared from CEF cultures, which were infected with viruses at a multiplicity of infection (MOI) of 0.01 and cultured for 72 h. Viruses were released by three freeze-th–w cycles (−70 °C and 37 °C) and stored at −70 °C for further use. Pfu or TCID_50_ titers were determined on CEFs according to the standard titration method [9, 31]. The BAC transfer vector plasmid pDEVgc-pHA2 was kindly provided by professor Niklaus Osterrieder from the Free University of Berlin [9]. The HA expression cassette containing a pMCMV IE promoter and a consensus HA gene (GenBank: KP019932) was synthesized and cloned into T-Vector pMD19 (Simple; Takara, Otsu, Japan) with slight modification to generate the plasmid pDEV-H5(UL55). Briefly, the HA gene was artificially synthesized based on a consensus sequence of the most updated HA genes of AIV clade 2.3.2.1 (GenBank: AB700635.1; JN986881.1; JN986882.1; JN646713.1; JN646716.1; HQ020376.1; CY098758.1; and JF975561.1) with a deletion of four basic amino acids at the cleavage site, as described previously [30]. The promoter pMCMV IE included a sequence complementary to the sequence between site 184336 and 182946 in the MCMV genome of (GenBank: GU305914.1) followed by a Kozak sequence. The plasmid pDEV-H5(UL55) KAN^in^ containing the HA expression cassette and a kanamycin resistance gene inserted at the *Sac* I restriction site was constructed by cutting and ligating for further *En Passant* recombination (Fig. 3).

### Cells, viral DNA extraction, and transfection

CEFs were propagated in Earle’s minimal essential medium (EMEM; Gibco, Los Angeles, CA USA) supplemented with 10 % newborn calf serum (NBCS; Gibco), 100 U/ml penicillin, and 100 μg/ml streptomycin at 37 °C under a 5 % CO_2_ atmosphere. Viral DNA was purified from infected cells by sodium dodecyl sulfate (SDS)-proteinase K extraction as described previously [35]. The transfection of DNA from plasmids, viruses, or BACs was achieved by calcium phosphate precipitation [35]. Briefly, approximately 200 ng DNA was mixed with water, and then 62 μl 2 M CaCl_2_ was added dropwise to a total volume of 500 μl. The transfection mixture was incubated over night at 4 °C followed by the addition of 500 μl cold 2 × HEPES-bufftered saline(HBS) solution dropwise. The medium in each well was replaced with 500 μl of fresh EMEM without NBCS or antibiotics and incubated with the transfection mixture at 37 °C for 3–4 h. Media were discarded and the plate was washed twice with PBS. 1.5 ml 15 % glycerol HBS solution was added to each well and the plate was incubated for 2 min. The transfection solution was replaced with EMEM supplemented with 10 % NBCS and antibiotics, after washing twice with PBS, for culture at 37 °C in an incubator with 5 % CO_2_.

### Multi-step growth kinetics

The growth characteristics of viruses were tested on primary or secondary CEFs with an MOI of 0.01 as described previously with a slight modification [9]. Briefly, the virus titers of the supernatant- and cell-associated viruses were checked at 0, 6, 12, 24, 36, 48, and 72 h p.i. for parental virus and mutants. For cell-associated viruses, infected cells were washed twice with PBS at each indicated time point and resuspended in 2 ml of EMEM for three freeze–thaw cycles to release viruses. Virus titers were tested following the standard pfu titration method [9] after removal of cellular residue by centrifugation at 500 × g for 10 min. To measure the titer of viruses in the supernatants, the supernatants of infected cell cultures were sampled at the indicated time points and titrated after removal of cellular debris by centrifugation. The growth kinetics curve was established based on data in three independent experiments, and the differences of titers at 48 h and 72 h p.i. were statistically analyzed by one-way analysis of variance (SPSS software package) [9].

### Bacterial manipulations

Electrocompetent *E. coli* cells were obtained from a commercial supplier (DH10B, Invitrogen) or prepared in our lab following previously described protocols [9] and GS1783, which was kindly provided by professor Nikolaus Osterrieder [9]. Electroporation was conducted exactly as described previously [36, 37]. Commercial chemical-competent *E. coli* cells DH5α (Takara) were used for chemical transformation of plasmid DNA as previously described [9]. DNA from the BAC or plasmid was prepared with a PureLink® Quick Plasmid Miniprep Kit (Invitrogen) and a Large–Construct Kit for midi-prep (Qiagen) according to the manufacturer’s instructions.

### PCR, restriction analysis, and sequencing

For the insertion of a kanamycin resistance gene into plasmid pDEV-H5(UL55), a pair of specific primers (Kan ins’ H5(HA) F and Kan ins’ H5(HA); Table 1) were designed with two *Sac* I restriction sites added to both terminals for cutting and ligation. The construct was examined by digestion with *Hind* III to check the correct insertion of the kanamycin resistance gene. Another pair of primers (DEV ins H5 casse UL55 F and DEV ins H5 casse UL55 R; Table 1) were used for insertion of the HA cassette into the DEV BAC clone through the *En Passant* protocol. To repair the gC genes of the gC-negative virus, a pair of primers (DEV gC flanking F and DEV gC flanking R; Table 1) were used to amplify a fragment that included the gC gene and two homologous 1 kpb flanking sequences of gC. The construct was sequenced using 20 specific primers (Table 1) to verify the sequence of the inserted HA expression cassette. The BACs and mutants were subjected to RFLP analysis with *EcoR* I and *Bam*H I, performed as described previously [9].

### Generation of a DEV^C-KCE^ infectious clone

A DEV^C-KCE^ infectious clone was generated with a method modified from the generation of BAC from the DEV 2085 strain [9]. Briefly, co-transfection of DNA from DEV^C-KCE^ and pDEVgc-pHA2 was conducted on primary CEFs (24 h) to allow for an insertion of mini-F sequences in lieu of gC. After green plaques were observed under UV light (488 nm), a homogeneous population of mini-F recombinant DEV^C-KCE^ (DEV^C-KCE^-miniF) was obtained by three rounds of picking and plating on CEFs and transferred into *E. coli* DH10B competent cells (Invitrogen) by electroporation. Positive clones with chloramphenicol resistance were examined through RFLP with *Eco*R I and *Bam*H I to select a clone of DEV^C-KCE^, which was then electroporated into *E. coli* GS1783 [9] for further genetic manipulation of the DEV genome. The resulting clone (pDEV^C-KCE^) was confirmed through RFLP. Next, gC was restored by homologous recombination as described previously [9]. A homogenous population of gC-recovered virus (DEV^C-KCE^gC^R^) was purified by picking and plating of the nonfluorescent plaques under UV light (488 nm) and verified by PCR and sequencing using a pair of primers (DEV gC flanking F and DEV gC flanking R; Table 1).

### Construction of a DEV-vectored HA

An HA expression cassette was inserted into the noncoding area between the ORFs UL55 and LORF11 in the DEV BAC clone pDEV^C-KCE^ genome to replace the nucleotide fragments between sites 263 and 291 (GenBank ID: EU082088.2) through the *En Passant* method [32] with minor modifications. Briefly, PCR was performed using plasmid pDEV-H5(UL55) KAN^in^ DNA as a template, and a pair of primers (DEV ins H5 casse UL55 F and DEV ins H5 casse UL55 R) to amplify the HA cassette with 40 bp homologous sequences flanking both terminals. After digestion with *Dpn* I to get rid of possible plasmid pollution, the PCR product was electroporated into competent pDEV^C-KCE^ cells to generate the first recombination with the cassette at the indicated sites. The target recombinant pDEV^C-KCE^-H5(UL55)-vectored HA clone was generated by deletion of the kanamycin resistance gene by the second recombination (Fig. 3). Selected clones without kanamycin resistance were confirmed by RFLP with *Eco*R I and *Bam*H I. The DEV^C-KCE^-vectored HA, termed DEV-H5(UL55), was generated with gC recovered in a way similar to that of DEV^C-KCE^gC^R^. The DEV^C-KCE^-H5(UL55) HA expression cassette was amplified by PCR (primers: DEV ins H5 casse UL55 F and DEV ins H5 casse UL55 R) and confirmed by sequencing with 20 specific sequencing primers (Table 1). DEV-H5(UL55) was subsequently cultured on CEFs for 20 generations to check the stability of the recombinant virus. F20 virus DNA was isolated for sequencing the HA expression cassette.

### IIF and western blotting

The expression DEV-vectored HA was examined by IIF and western blotting as previously described [9]. For IIF, DEV-H5(UL55) F20 was inoculated onto primary or secondary CEFs with a ratio of 50–100 pfu per well on a six-well plate. At 48 h after inoculation, the infected cells were fixed with cold fixing solution (ethanol (96 %): acetone = 3:1) for 20 min at −20 °C. The fixing solution wad discarded, and the cells were washed once with PBS and then permeabilized in PBS with 0.1 % Triton X-100 for 5 min. After fixation, a PBS solution with 3 % BSA was added to block the sample wells for 1 h or overnight. A mixture of monoclonal antibodies against AIV H5N1 HA (Genescript, Nanjing, China) was added and the samples were incubated for 1 h at room temperature. Each well was washed three times with PBS, a 1:2000 solution of goat-anti-mouse IgG antibodies conjugated with Alexa488 (Invitrogen) was added, and the samples were incubated for 1 h, then observed under UV light (488 nm).

For western blotting, CEFs were infected with F5 or F20 DEV-H5(UL55) viruses with an MOI of 0.01. Infected cells were lysed and cell lysates were denatured by heating at 95 °C for 10 min. Proteins were separated by SDS-10 % polyacrylamide gel electrophoresis (PAGE), and then transferred to nitrocellulose membranes (Merck) as described previously [11]. A mixture of H5 monoclonal antibodies (Genescript) was used as the primary antibodies for western blotting and a 1:10,000 dilution of goat-anti-mouse IgG (ABcom) was used as the secondary antibody. DEV^C-KCE^-infected CEFs were used as a control. Samples were detected with enhanced chemiluminescence (Sigma-Aldrich).

### Safety and immunogenicity of DEV-vectored HA in ducks and chickens

For the duck plague protection test, a total of 40 commercial ducks (5 weeks old) were randomly divided into four groups (Table 2). Ducks in groups A-DP(5D) and B-DP(5D) were inoculated intramuscularly with DEV-H5(UL55) virus and DEV vaccine (commercial product from Nanjing Tech-bank Bio-industry Co., Ltd), respectively, each at a dose of 1 × 10^6^ TCID_50_. Ducks in groups C-DP(5D) and D-DP(5D) were inoculated intramuscularly with 0.2 ml of PBS as controls. Three weeks after inoculation, all the groups except group D-DP(5D) were challenged with virulent DEV at a dose of 100 LD_50_ and observed daily for 14 days to analyze the morbidity and mortality.

**Table 2:** Groups of animals tested to evaluate the safety, efficacy, and immunogenicity of DEV-H5(UL55)

Group	Vaccine or PBS	Dose	Animal/age
A-DP(5D)	DEV-H5(UL55)	1 × 10^6^ TCID_50_, 0.2 mL	Ducks/5w^a^
B-DP(5D)	DEV vaccine	1 × 10^6^ TCID_50_, 0.2 mL	Ducks/5w
C-DP(5D)	PBS	0.2 mL	Ducks/5w
D-DP(5D)	PBS	0.2 mL	Ducks/5w
A-AI(2D)	DEV-H5(UL55)	1 × 10^7^ TCID_50_, 0.2 mL	Ducks/2w^b^
B-AI(2D)	DEV-H5(UL55)	1 × 10^6^ TCID_50_, 0.2 mL	Ducks/2w
C-AI(2D)	DEV-H5(UL55)	1 × 10^5^ TCID_50_, 0.2 mL	Ducks/2w
D-AI(2D)	Inactivated AI(H5N1) Re-6 vaccine	0.5 mL and 1 mL at 2w and 5w respectively	Ducks/2w
E-AI(2D)	PBS	0.2 mL	Ducks/2w
A-AI(1C)	DEV-H5(UL55)	1 × 10^7^ TCID_50_, 0.2 mL	SPF chickens/1w^c^
B-AI(1C)	DEV-H5(UL55)	1 × 10^6^ TCID_50_, 0.2 mL	SPF chickens/1w
C-AI(1C)	DEV-H5(UL55)	1 × 10^5^ TCID_50_, 0.2 mL	SPF chickens/1w
D-AI(1C)	Inactivated AI(H5N1) Re-6 vaccine	0.3 mL	SPF chickens/1w
E-AI(1C)	PBS	0.2 mL	SPF chickens/1w
A-AI(3C)	DEV-H5(UL55)	1 × 10^7^ TCID_50_, 0.2 mL	Layers/3w^d^
B-AI(3C)	DEV-H5(UL55)	1 × 10^6^ TCID_50_, 0.2 mL	Layers/3w
C-AI(3C)	DEV-H5(UL55)	1 × 10^5^ TCID_50_, 0.2 mL	Layers/3w
D-AI(3C)	Inactivated AI(H5N1) Re-6 vaccine	0.3 mL	Layers/3w
E-AI(3C)	PBS	0.2 mL	Layers/3w

^a^A total of 40 5-week-old commercial ducks were randomly divided into four groups of A-DP(5D), B-DP(5D), C-DP(5D), and D-DP(5D) to test the safety and protection efficiency of DEV-H5(UL55) against virulent DEV challenge

^b^A total of 50 2-week-old commercial ducks were randomly divided into five groups of A-AI(2D), B-AI(2D), C-AI(2D), D-AI(2D), and E-AI(2D) to test of safety and potency of DEV-H5(UL55) in stimulation of HI antibodies against AI(H5N1), compared with inactivated AI(H5N1) Re-6 vaccine

^c^A total of 50 1-week-old SPF chickens were randomly divided into five groups of A-AI(1C), B-AI(1C), C-AI(1C), D-AI(1C), and E-AI(1C) to test the safety and potency of DEV-H5(UL55) in the stimulation of HI antibodies against AI(H5N1)

^d^A total of 50 3-week-old commercial layer chickens were randomly divided into five groups of A-AI(3C), B-AI(3C), C-AI(3C), D-AI(3C), and E-AI(3C) to test the safety and potency of DEV-H5(UL55) in the stimulation of HI antibodies against AI(H5N1)

To assess the immunogenicity of the DEV-vectored HA against AIV in ducks, 50 ducks (2 weeks old) were randomly divided into five groups: A-AI(2D), B-AI(2D), C-AI(2D), D-AI(2D), and E-AI(2D) (Table 2). Ducks in groups A-AI(2D), B-AI(2D), and C-AI(2D) were vaccinated intramuscularly with DEV-H5(UL55) at a dose of 1 × 10^7^, 1 × 10^6^, and 1 × 10^5^ TCID_50_, respectively. Ducks in group D-AI(2D) were inoculated subcutaneously with 0.5 ml and 1 ml inactivated AIV H5N1 Re-6 vaccine from a commercial supplier (QYH, Zhengzhou, China) at 2 and 5 weeks of age, respectively. Ducks in the control group E-AI(2D) were inoculated with 0.2 ml of PBS. Serum samples from all ducks were collected prior to inoculation and at 1, 2, 3, 4, 5, and 6 weeks post-immunization to test the HI titers with kits (HWBD, Harbin China) following the manufacturer’s instructions.

To evaluate the safety and immunogenicity of the DEV-vectored HA against AIV for chickens, the HI antibody levels were assessed in 50 1-week-old SPF chickens and 50 3-week-old commercial layer chickens (Table 2). SPF chickens were randomly divided into five groups: A-AI(1C), B-AI(1C), C-AI(1C), D-AI(1C), and E-AI(1C). Commercial layer chickens were also divided into five groups: A-AI(3C), B-AI(3C), C-AI(3C), D-AI(3C), and E-AI(3C). In groups A-AI(1C)/A-AI(3C), B-AI(1C)/B-AI(3C), and C-AI(1C)/C-AI(3C), chickens were vaccinated intramuscularly with DEV-H5(UL55) at a dose of 1 × 10^7^, 1 × 10^6^, and 1 × 10^5^ TCID_50_, respectively. Chickens in groups D-AI(1C)/D-AI(3C) were inoculated subcutaneously with 0.3 ml of inactivated AIV H5N1 Re-6 vaccine (QYH) according to the manufacturer’s instructions. Chickens in control groups E-AI(1C)/E-AI(3C) were inoculated with 0.2 ml of PBS. Serum samples from all chickens were collected prior to inoculation and at 1, 2, 3, 4, 5, and 6 weeks post-vaccination and the HI titers of these samples were measured. All birds were monitored for clinical signs throughout the experiments.

All animal studies were approved by the Institutional Animal Care and Use Committee and were conducted following the guidelines of the Institutional Biosafety Committee at the Jiangsu Academy of Agriculture Sciences. Experiments involving virulent DEV were conducted under Biosafety Level 2+ containment.
